# Electrocardiographic Findings in Children With Growth Hormone Deficiency

**DOI:** 10.7759/cureus.36385

**Published:** 2023-03-20

**Authors:** Münevver Yılmaz, Dolunay Gürses, Aysun Ata

**Affiliations:** 1 Pediatric Cardiology, Faculty of Medicine, Pamukkale University, Denizli, TUR; 2 Pediatric Endocrinology, Adana City Training and Research Hospital, Adana, TUR

**Keywords:** tp-e/qtcmax ratio, tp-e/qtmax ratio, tp-e interval, electrocardiography (ecg), children, human growth hormone deficiency

## Abstract

Introduction

It has been shown that cardiac functions begin to deteriorate in growth hormone (GH) deficiency even in childhood. However, little is known about how GH deficiency affects arrhythmogenesis. The aim of this study was to evaluate the parameters of P wave dispersion (Pd), QT dispersion (QTd), corrected QT (QTc) dispersion (QTcd), T wave peak-to-end (Tp-e) interval, Tp-e/QT ratio, and Tp-e/QTc ratio in children with GH deficiency. This study also aimed to evaluate the relationship of these parameters with insulin-like growth factor 1 (IGF-1) and insulin-like growth factor binding protein 3 (IGFBP-3).

Method

In the study, records of children diagnosed with GH deficiency in Adana City Training and Research Hospital Pediatric Endocrine Outpatient Clinic between September 2021 and December 2022 were retrospectively reviewed. The control group consisted of children in the same age group who applied to the Emergency Outpatient Clinic with a complaint of chest pain and no pathological finding was detected. The electrocardiograms (ECGs) of all patients were retrospectively evaluated.

Results

There were a total of 82 children in the study, 41 of whom were diagnosed with GH deficiency and 41 in the healthy control group. The age and male/female ratio of children with GH deficiency were similar to those in the control group (p>0.05). There were 27 (66%) children with complete GH deficiency and 14 (34%) children with partial GH deficiency. P wave dispersion was similar in both GH-deficient children and control group children. It was also similar in children with complete and partial GH deficiency (p>0.05). QT and QTc dispersions were found to be increased in children with GH deficiency, although not statistically significant, compared to the control group (p>0.05). Tp-e interval, Tp-e/QTmax (longest QT interval), and Tp-e/QTcmax (longest QTc interval) ratios were increased in children with GH deficiency compared to the control group (p=0.001, p=0.003, and p=0.001, respectively). QT and QTc dispersion, Tp-e interval, Tp-e/QTmax, and Tp-e/QTcmax ratios were found to be increased in children with complete GH deficiency compared to children with partial GH deficiency, but the difference was not significant (p>0.05). No correlation was found between these ECG parameters and IGF-1, IGFBP-3, and peak GH levels after stimulation tests (p>0.05).

Conclusion

We found in our study that the Tp-e interval was longer and Tp-e/QT and Tp-e/QTc ratios were increased in children with GH deficiency. These results suggest that the risk of ventricular arrhythmias in children with GH deficiency may start to increase from childhood. However, further prospective studies are needed to confirm our results.

## Introduction

Growth hormone (GH) has effects on many systems as well as stimulating growth in children. In addition to cardiac growth in the cardiovascular system, it plays a role in the regulation of cardiac structure and function [1]. Both GH and insulin-like growth factor 1 (IGF-1) receptors are expressed in the myocardium, and IGF-1 has been shown to stimulate myofibril development and increase isometric strength [2,3]. Less than 1% of total serum IGF-1 is freely circulating, most of it is bound to insulin-like growth factor binding proteins (IGFBPs). IGFBPs have IGF-dependent and IGF-independent functions [4,5]. Of the six different circulating IGFBPs, IGFBP-3 is the most abundant, and it has been shown that low IGFBP-3 levels are associated with an increased risk of cardiovascular disease [4,6].

Growth hormone deficiency is characterized by significant growth retardation, slowing of growth rate, retardation in bone age, and low GH level despite spontaneous and pharmacological stimulation, when there is no other reason to explain the short stature [7,8]. It is known that GH deficiency affects sympathetic activity and has negative effects on the structure and function of the cardiovascular system. GH deficiency can cause decreased left ventricular mass and ejection fraction, resulting in decreased cardiac performance in adults. It has been shown that cardiac functions are affected even in childhood in GH deficiency and begin to improve after 12 months of GH treatment [1,7,9]. However, little is known about how GH deficiency affects arrhythmogenesis [1,10].

In experimental studies, it has been reported that GH exerts cytoprotective effects after coronary artery occlusion and reduces local myocardial norepinephrine release and arrhythmogenesis [11]. In studies with adults, heart rate variability was evaluated in patients with GH deficiency, and in studies using both frequency domain and time domain parameters, impaired response to sympathetic activation has been demonstrated [12,13]. There is only one study evaluating heart rate variability in children with GH deficiency, and global heart rate variability was found to be reduced in this study [14].

A surface electrocardiogram (ECG) is an easily accessible test that provides a rapid assessment of cardiac electrophysiology and can be used to assess arrhythmic risk [15]. In the assessment of arrhythmia risk, QT dispersion (QTd) for myocardial conduction disturbances, heart rate corrected QT (QTc) dispersion (QTcd), and P wave dispersion (Pd) for atrial conduction variability are used. In addition, in recent years, the interval from the peak to the end of the T wave (Tp-e interval), Tp-e/QT ratio, and Tp-e/QTc ratio have also been defined as predictive electrocardiographic markers for ventricular arrhythmias [16-20]. There are only two studies in the literature evaluating ECG in children with GH deficiency. In the study of Alkan et al. [21], in which they evaluated ECG parameters in children with complete and partial GH deficiency and in the control group, changes were found in the P wave and QTc dispersion on the ECG between the groups. On the other hand, Nygren et al. [22] showed that the QTc interval did not change before and after GH treatment.

The aim of this study was to evaluate the parameters of P wave dispersion, QT dispersion, QTc dispersion, Tp-e interval, Tp-e/QT ratio, and Tp-e/QTc ratio in children with GH deficiency. In addition, the study is also aimed at evaluating the relationship of these parameters with IGF-1 and IGFBP-3.

## Materials and methods

In the study, records of children diagnosed with GH deficiency in Adana City Training and Research Hospital Pediatric Endocrine Outpatient Clinic between September 2021 and December 2022 were retrospectively reviewed. GH deficiency was diagnosed according to clinical and auxology criteria. The diagnosis of GH deficiency was considered in a child with short stature (height below 3 standard deviation (SD) or less than 2 SD and annual growth rate below 2 SD) after excluding other causes of growth retardation (hypothyroidism, chronic systemic disease, Turner syndrome, or skeletal system disorders). GH provocation tests (L-dopa and insulin tolerance test) were performed. A peak GH value of <10 µg/L after two separate GH provocation tests was defined as GH deficiency [8]. Children with GH deficiency were grouped after two provocation tests as children with a peak GH level of <7 µg/L (complete GH deficiency) and those with a peak GH level of 7-10 µg/L (partial GH deficiency). Patients diagnosed with GH deficiency and whose ECG was taken after provocation tests were included in the study. Those with pituitary hormone deficiency other than GH and those with pathological findings in pituitary magnetic resonance imaging were excluded from the study. Children in the same age group, who presented to the Pediatric Emergency Outpatient Clinic with the complaint of chest pain, whose ECG was recorded and no pathological cause was found, were included in the study. This group consisted of healthy children with idiopathic chest pain whose history, physical examination, laboratory, and imaging revealed no pathology. First-degree relatives who would cause changes in ECG parameters in the family and those with a history of drug use that could cause changes in ECG parameters were excluded from the study, both in the GH deficiency group and in the control group. The ECG recordings of the patients in the control group and GH deficiency group were reviewed retrospectively by the same pediatric cardiologist.

ECG recordings were made at a rate of 25 mm/second, with an amplitude of 1 mV, and with 12 standard leads containing at least six QRS complexes for each lead. Measurements in all leads were performed manually. The difference between the longest P wave (Pmax) and the shortest P wave (Pmin) was considered as the P dispersion (Pd=Pmax-Pmin). The time between the onset of the QRS complex and the point where the descending branch of the T wave cut the isoelectric segment was taken as the QT interval. Leads in which T wave could not be detected were excluded. QT dispersion was defined as the difference between the longest (QTmax) and the shortest (QTmin) QT interval (QTd=QTmax-QTmin). Bazett’s formula (QTc=QT/√RR) was used for QTc. QTc dispersion was similarly determined as the difference between the longest QTc (QTcmax) interval and the shortest QTc (QTcmin) interval. The Tp-e interval was defined as the interval from the peak of the T wave to the end of the T wave. Tp-e interval measurements were made from lead V5. In addition, Tp-e/QTmax and Tp-e/QTcmax ratios were calculated.

Approval was obtained from the Non-Invasive Clinical Research Ethics Committee of Pamukkale University (10.01.2023/01) before conducting the study.

The Statistical Package for the Social Sciences (SPSS) for Windows version 20 (IBM SPSS Statistics, Armonk, NY, USA) was used for the statistical evaluation of data. Continuous variables were given as mean±standard deviation (minimum-maximum), and categorical variables were given as number (percentage). Kolmogorov-Smirnov and Shapiro-Wilk tests were used to determine the normal distribution. In the comparison of independent data, Student’s t-test was used for parametric data and the chi-square test was used for the comparison of categorical variables. The correlation of continuous variables was evaluated using Pearson correlation analysis, and p<0.05 was accepted as statistically significant.

## Results

A total of 82 children were included in the study, 41 of whom were diagnosed with GH deficiency and 41 in the healthy control group. When demographic findings are evaluated, the age and male/female ratio of the children with GH deficiency were similar to the children in the control group (p>0.05) (Table 1). The mean height, body weight, and body mass index were 123±18.4 (86.5-153.8) cm, 26.1±9.7 (11.2-59) kg, and 16.6±2.2 (12.8-24.9) kg/m^2^, respectively, in children with GH deficiency. On the other hand, these values were 140.5±21.7 (100-184) cm, 37.9±16.7 (15-74) kg, and 17.9±3.5 (13.2-25.51) kg/m^2^, respectively, in the control group. The height and body weight of children with GH deficiency were significantly lower than the control group (p=0.001), and the body mass index was at the low threshold (p=0.05). While height standard deviation score (SDS) and body weight SDS were significantly lower in children with GH deficiency (p<0.001), no significant difference was found in body mass index SDS (p=0.074) (Table 1). The peak GH levels of children with GH deficiency after IGF-1, IGFBP-3, and provocation tests are shown in Table 1.

**Table 1:** Demographic, laboratory, and ECG data of the study group. ECG: electrocardiogram, SD: standard deviation, SDS: standard deviation scores, IGF-1: insulin-like growth factor 1, IGFBP-3: insulin-like growth factor binding protein 3, PGH: peak growth hormone, HR: heart rate, Pmax: longest P wave, Pmin: shortest P wave, Pd: P wave dispersion, QTmax: longest QT interval, QTmin: shortest QT interval, QTd: QT dispersion, QTcmax: longest QTc interval, QTcmin: shortest QTc interval, QTcd: corrected QT dispersion, max: maximum, min: minimum, Tp-e: T wave peak-to-end

Mean±SD (min-max)	Patients with growth hormone deficiency (n=41)	Control group (n=41)	p value
Sex (male/female)	26/15	27/14	0.817
Age (years)	9.75±3.2 (4-15)	9.78±3.2 (4-15)	0.972
Height SDS	-2.67±0.86 (-5.77-(-2))	0.29±1.2 (-1.9-1.92)	0.001
Weight SDS	-1.89±0.85 (-3.31-(-0.6))	0.13±1.1 (1.8-1.94)	0.001
Body mass index SDS	-0.56±1 (-2.63-1.13)	-0.11±1.14 (-1.8-1.57)	0.074
IGF-1 (µg/mL)	116.8±64 (20.3-334)	-	
IGFBP-3 (ng/mL)	20.3±44.9 (1.86-140)	-	
PGH with L-dopa stimulation test (µg/L)	5.2±3 (0.1-9.9)	-	
PGH with insulin stimulation test (µg/L)	3.1±2.6 (0.1-9.9)	-	
HR (bpm)	84.7±16.4 (56-115)	92.1±21.4 (58-154)	0.082
Pmax (ms)	80.8±14.6 (44-120)	84.1±8.6 (66-110)	0.208
Pmin (ms)	59.5±14.4 (36-90)	63.2±9.1 (42-90)	0.161
Pd (ms)	21.3±7.3 (8-40)	20.9±4.5 (12-30)	0.798
QTmax (ms)	344.8±32.5 (280-420)	342.1±32.5 (275-400)	0.71
QTmin (ms)	314.6±36.5 (240-380)	313.4±30.7 (240-360)	0.87
QTd (ms)	30.1±12.7 (10-60)	28.7±9.7 (10-40)	0.559
QTcmax (ms)	408.2±13 (360-425)	410±13.7 (365-423)	0.474
QTcmin (ms)	360±21.3 (329-406)	369±24.5 (320-423)	0.065
QTcd (ms)	48.3±21.5 (5-88)	41±24.5 (5-90)	0.153
Tp-e (ms)	71.8±10.3 (52-90)	64.7±8.3 (48-80)	0.001
Tp-e/QTmax	0.21±0.03 (0.15-0.28)	0.19±0.02 (0.15-0.25)	0.003
Tp-e/QTcmax	0.18±0.02 (0.13-0.22)	0.16±0.02 (0.12-0.20)	0.001

When ECG parameters are compared, no significant difference was found in heart rates between children with GH deficiency and in the control group (p>0.05). The maximum and minimum P wave intervals and P dispersion were similar in children with GH deficiency and in the control group (p>0.05) (Table 1). There was no significant difference between the groups in terms of maximum and minimum QT and QTc durations (p>0.05). Both QT and QTc dispersions were increased in children with GH deficiency compared to the control group, but the difference was not statistically significant (p>0.05) (Table 1). When we evaluate the Tp-e interval, both Tp-e/QTmax and Tp-e/QTcmax ratios with Tp-e interval were found to be significantly increased in children with GH deficiency compared to the control group (p=0.001, p=0.003, and p=0.001, respectively).

When the children with GH deficiency were divided into two groups according to the results of the provocation tests, there were 27 (66%) children with complete GH deficiency and 14 (34%) children with partial GH deficiency. There was no significant difference between children with complete and partial GH deficiency in terms of age, gender, height SDS, body weight SDS, and body mass index SDS (p>0.05), and heart rates were also found to be similar (p>0.05) (Table 2). There was no significant difference between children with complete and partial GH deficiency in terms of maximum P wave duration, minimum P wave duration, P wave dispersion, and maximum and minimum QT and QTc intervals (p>0.05). QT dispersion, QTc dispersion, Tp-e interval, Tp-e/QTmax ratio, and Tp-e/QTcmax ratio were increased in children with complete GH deficiency compared to children with partial GH deficiency. However, this difference between groups was not statistically significant (p>0.05) (Table 2). In addition, P wave, QT and QTc dispersion, Tp-e interval, Tp-e/QTmax, and Tp-e/QTcmax ratios were not significantly related to peak GH levels after IGF-1, IGFBP, and GH peak GH levels after stimulation tests (p>0.05) (Table 3).

**Table 2:** Demographic, laboratory, and ECG data in complete and partial growth hormone deficiency groups. ECG: electrocardiogram, SD: standard deviation, SDS: standard deviation scores, IGF-1: insulin-like growth factor 1, IGFBP-3: insulin-like growth factor binding protein 3, HR: heart rate, Pmax: longest P wave, Pmin: shortest P wave, Pd: P wave dispersion, QTmax: longest QT interval, QTmin: shortest QT interval, QTd: QT dispersion, QTcmax: longest QTc interval, QTcmin: shortest QTc interval, QTcd: corrected QT dispersion, max: maximum, min: minimum, Tp-e: T wave peak-to-end

Mean±SD (min-max)	Complete growth hormone deficiency group (n=27)	Partial growth hormone deficiency group (n=14)	p value
Sex (male/female)	18/9	8/6	0.548
Age (years)	9.63±3 (4-15)	10±3.7 (5-14)	0.729
Height SDS	-2.8±0.8 (-5.77-(-2))	-2.44±0.7 (-4.18-(-2))	0.279
Weight SDS	-1.78±0.8 (-3.31-(-0.6))	-2.1±1 (-3.2-(-0.7))	0.229
Body mass index SDS	-0.35±1 (-2.63-1.13)	-1.03±1.14 (-2.24-0.92)	0.06
IGF-1 (µg/mL)	106.6±54.5 (20.3-197)	136.5± 77.8 (31.2-334)	0.159
IGFBP-3 (ng/mL)	23.7±49.1 (1.86-140)	13.9±36.3 (2.2-140)	0.518
HR (bpm)	84.2±16.4 (56-115)	85.6±14.8 (60-105)	0.791
Pmax (ms)	78.3±13 (44-100)	85.6±16.8 (66-120)	0.133
Pmin (ms)	57.9±13.8 (36-80)	62.5±15.5 (40-90)	0.341
Pd (ms)	20.4±7.1 (8-40)	23.1±7.5 (12-30)	0.264
QTmax (ms)	345.7±37.7 (280-420)	342.9±29.2 (300-400)	0.792
QTmin (ms)	314.4±37.7 (240-380)	315±35.7 (280-380)	0.964
QTd (ms)	31.3±13 (10-50)	27.9±13.7 (10-60)	0.417
QTcmax (ms)	409.4±14 (360-425)	406±12.3 (385-425)	0.438
QTcmin (ms)	358±21.5 (329-402)	363.3±21.3 (3230-406)	0.465
QTcd (ms)	51.3±21.2 (13-88)	42.6±21.5 (5-73)	0.225
Tp-e (ms)	73.8±9.1 (58-90)	67.9±11.6 (52-84)	0.08
Tp-e/QTmax	0.22±0.03 (0.17-0.28)	0.2±0.03 (0.15-0.25)	0.084
Tp-e/QTcmax	0.18±0.02 (0.14-0.22)	0.17±0.03 (0.13-0.21)	0.072

**Table 3:** Correlation of IGF-1, IGFBP-3, and PBH levels and ECG parameters. ECG: electrocardiogram, IGF-1: insulin-like growth factor 1, IGFBP-3: insulin-like growth factor binding protein 3, PGH: peak growth hormone, Pd: P wave dispersion, QTd: QT dispersion, QTcd: corrected QT dispersion, max: maximum, min: minimum

Laboratory parameters		Pd (ms)	QTd (ms)	QTcd (ms)	Tp-e (ms)	Tp-e/Qtmax (ms)	Tp-e/Qtcmax (ms)
IGF-1 (µg/mL)	r	-0.009	-0.047	-0.007	-0.038	-0.115	-0.017
	p value	0.958	0.771	0.963	0.812	0.472	0.915
IGFBP-3 (ng/mL)	r	-0.223	-0.183	-0.097	0.063	-0.06	0.077
	p value	0.162	0.251	0.546	0.697	0.711	0.631
PGH with L-dopa stimulation test (µg/L)	r	0.203	-0.250	-0.276	-0.077	-0.187	-0.055
	p value	0.203	0.115	0.081	0.632	0.241	0.733
PGH with insulin stimulation test (µg/L)	r	-0.188	-0.096	-0.153	-0.184	-0.154	-0.228
	p value	0.320	0.615	0.418	0.330	0.416	0.226

## Discussion

In this study, we evaluated ECG findings in children with GH deficiency, and we found that the Tp-e interval, Tp-e/QTmax, and Tp-e/QTcmax ratios were increased in children with GH deficiency compared to the healthy control group.

In adults, both GH excess and deficiency have been associated with an increased risk of cardiac arrhythmias, including atrial fibrillation (AF), and structural remodeling of the left atrium has been demonstrated that may mediate the increased risk of AF in patients with GH deficiency [1,23,24]. Pd is a strong marker of the heterogeneous spread of anatomical remodeling and activation in the atrium. It has been suggested that Pd, believed to reflect non-homogeneous atrial conduction, is useful in determining the risk of supraventricular arrhythmias, particularly paroxysmal AF [18,19,25]. In the study of Alkan et al. [21], in which they evaluated 47 children with GH deficiency (30 complete GH deficiency and 17 partial GH deficiency), the P wave interval was found to be shorter in children with partial GH deficiency. On the other hand, Pd was found to be similar in children with complete and partial GH deficiency and in the healthy control group. In our study, there was no difference in terms of Pd in children with GH deficiency and in the healthy control group. In addition, Pd was similar in children with complete and partial GH deficiency. No correlation was found between Pd and peak GH levels after IGF-1, IGFBP-3, and provocation tests.

Experimental studies have shown that IGF-1 regulates sarcolemmal potassium channel activity and late sodium current in rat cardiomyocytes, thereby affecting cardiac repolarization and QTc [26,27]. In addition, in a sample of elderly people aged 60-64 years, decreased levels of IGF-1 were associated with prolonged QTc interval [4]. Long QT and QTc intervals and increased QT and QTc dispersions are well-known risk factors for all-cause mortality and morbidity [15,28]. In the study of Alkan et al. [21], although QTc and QT dispersion were longer in children with GH deficiency compared to the control group, no statistically significant difference was found. Nygren et al. [22], in their study evaluating 89 children with GH deficiency, reported that the QTc interval did not change before and after GH treatment. In our study, QT and QTc dispersions were increased in children with GH deficiency and in the control group, although not statistically significant. When patients with complete and partial GH deficiency are evaluated, QT and QTc dispersions were again increased in children with complete GH deficiency, although not statistically significant. QTd and QTcd were found to be not correlated with the peak level of GH after IGF-1, IGFBP-3, and provocation tests.

IGF-1 is thought to protect cardiac myocytes from arrhythmogenesis and apoptosis by activating PI3K/Akt intracellular signal transduction [26]. Changes in T wave shape or duration reflect the heterogeneity of ventricular repolarization, and prolonged Tp-e interval on ECG indicates repolarization heterogeneity [17]. It is argued that an abnormally prolonged Tp-e interval on ECG is a risk factor for ventricular arrhythmic mortality and all-cause mortality, independent of age, gender, comorbidities, QRS interval, and corrected QT interval [16]. It has been suggested that the Tp-e/QTc ratio is a better predictor of ventricular repolarization because of the interaction between QT and heart rate and body weight [17,20]. Tp-e interval and Tp-e/QT ratios have been investigated in many diseases that affect the cardiovascular system, such as diabetes and hypothyroidism, and have been reported to be risk factors for ventricular arrhythmias [29,30]. Alkan et al. [21] found a longer Tp-e interval in children with complete GH deficiency compared to children with partial GH deficiency and the control group. In the same study, although the Tp-e/QTc ratio was higher in children with complete GH deficiency, no statistically significant difference was found. In our study, Tp-e interval, Tp-e/QTmax, and Tp-e/QTcmax ratios were increased in children with GH deficiency compared to the control group. Although the same parameters were found to be increased in children with complete GH deficiency compared to children with partial GH deficiency, the difference was not significant. No correlation was found between these ECG parameters and peak GH levels after IGF-1, IGFBP-3, and peak GH levels after stimulation tests. The increase in Tp-e interval, Tp-e/QTmax, and Tp-e/QTcmax ratios suggests that there may be an increased risk of arrhythmia in children with GH deficiency. Therefore, we think that ECG monitoring is important in these children.

The main limitation of this study is that it is a retrospective study with a relatively small sample size. Other limitations are the lack of echocardiographic evaluation and evaluation of children with GH deficiency after treatment. Therefore, further prospective studies with larger populations are needed to confirm our results.

## Conclusions

We found in our study that the Tp-e interval is longer and the Tp-e/QT and Tp-e/QTc ratios are increased in children with GH deficiency. These results suggest that the risk of ventricular arrhythmias in children with GH deficiency may start to increase from childhood. However, further prospective studies are needed to confirm our results.
